# Gene regulatory network stabilized by pervasive weak repressions: microRNA functions revealed by the May–Wigner theory

**DOI:** 10.1093/nsr/nwz076

**Published:** 2019-07-25

**Authors:** Yuxin Chen, Yang Shen, Pei Lin, Ding Tong, Yixin Zhao, Stefano Allesina, Xu Shen, Chung-I Wu

**Affiliations:** 1 School of Life Science, Sun Yat-Sen University, Guangzhou 510275, China; 2 Target Discovery Research, Boehringer Ingelheim Pharma GmbH & Co KG, 88397 Biberach an der Riß, Germany; 3 Department of Biostatistics, School of Public Health, Yale University, New Haven, CT 06520, UK; 4 Department of Ecology and Evolution, University of Chicago, Chicago, IL 60637, UK

**Keywords:** microRNAs, network stability, canalization, May–Wigner theory, systems biology, RNA crosstalk

## Abstract

Food web and gene regulatory networks (GRNs) are large biological networks, both of which can be analyzed using the May–Wigner theory. According to the theory, networks as large as mammalian GRNs would require dedicated gene products for stabilization. We propose that microRNAs (miRNAs) are those products. More than 30% of genes are repressed by miRNAs, but most repressions are too weak to have a phenotypic consequence. The theory shows that (i) weak repressions cumulatively enhance the stability of GRNs, and (ii) broad and weak repressions confer greater stability than a few strong ones. Hence, the diffuse actions of miRNAs in mammalian cells appear to function mainly in stabilizing GRNs. The postulated link between mRNA repression and GRN stability can be seen in a different light in yeast, which do not have miRNAs. Yeast cells rely on non-specific RNA nucleases to strongly degrade mRNAs for GRN stability. The strategy is suited to GRNs of small and rapidly dividing yeast cells, but not the larger mammalian cells. In conclusion, the May–Wigner theory, supplanting the analysis of small motifs, provides a mathematical solution to GRN stability, thus linking miRNAs explicitly to ‘developmental canalization’.

## INTRODUCTION

Large networks characterize many biological systems. Of particular interest to ecologists and evolutionists are food webs and gene regulatory networks (GRNs). These large and highly connected networks are intrinsically unstable according to the May–Wigner theory [1]. Stability is defined as the speed at which every node would return to the equilibrium after perturbation. Hence, in large networks, the probability that all nodes would return to the equilibrium is diminished. The issue of stability has been extensively analyzed for food webs [1–3] but the underlying mathematics of stabilization should be common among networks.

Stability of GRNs is an especially challenging problem for several reasons. First, GRNs are typically large with thousands of nodes, each representing the abundance of an mRNA. Size alone may demand that GRNs evolve mechanisms for stabilization. Second, there are as many types of GRNs as there are tissues in multicellular organisms. The stabilizing mechanism thus needs to be general. Third, unlike food webs that may tolerate substantial fluctuations in node values (i.e. species abundance), GRNs can only function within a small range of transcript abundance as transcriptome data show. Fourth, and perhaps most importantly, GRN stability may underlie the ‘developmental canalization’ proposed by Waddington [4].

Canalization is a metaphor for water traveling along canals [4]. Ever perturbed constantly, water always returns to the canal and flows along a predetermined path. There are two types of motion: (i) flowing along the path and (ii) returning to the canal quickly after perturbation. These two motions occur in very different timescales and canalization (in the narrow sense) refers to the second motion. GRN canalization means the quick return to the developmental path after the network is perturbed, for example, by cell divisions.

Although ‘canalized development’ originally referred to phenotypic stability, every level of network subsumed under the phenotype—including the metabolome, proteome and transcriptome—should be stable as well. A stable GRN, measured by the transcriptome, is hence necessary for developmental canalization and could be the first step in the process. For GRN stability, microRNAs (miRNAs) have been postulated to be the canalizing molecules [4–9]. These are a very large class of small regulatory RNAs that degrade mRNAs and repress translation, the former being particularly important [10].

While substantial literature have implicated a role for miRNAs in canalization [11–14], a rigorous theory that connects known miRNA actions to GRN stability is still absent. Many analyses have been carried out on small motifs of 2–5 nodes [5,9], but motifs cannot be easily expanded into a network. For example, adding only one extra node to a coherent motif can make it incoherent, and vice versa [7]. An alternative approach is the large RNA:RNA networks [15,16], which at present cannot address the stability issue. In this study, we provide a mathematical solution to GRN stability by applying the May–Wigner theory to the empirical data on miRNA activities.

## RESULTS

### Diffuse actions of miRNAs

For regulatory genes, miRNAs seem paradoxical for two reasons: (i) exclusive downregulation of their direct targets, and (ii) broad and weak repression of hundreds of target genes. In the conventional view, miRNAs repress targets in order to effect phenotypic changes, but that view is contradicted by their peculiar properties presented above [17]. By analyzing multiple miRNA targets and phenotypes concurrently, Liufu *et al*. [18] recently concluded that the role of miRNAs is indeed in minimizing phenotypic fluctuation (i.e. canalization) rather than effecting directional changes, as has been heatedly debated [17,19,20].

We first present the defining characteristics of miRNAs, i.e. weak and broad repression of mRNAs. Unlike previous analyses [21–29], this study pays special attention to weak interactions, which will later be subjected to mathematical interpretation.

#### Number of targets

**Figure 1.**
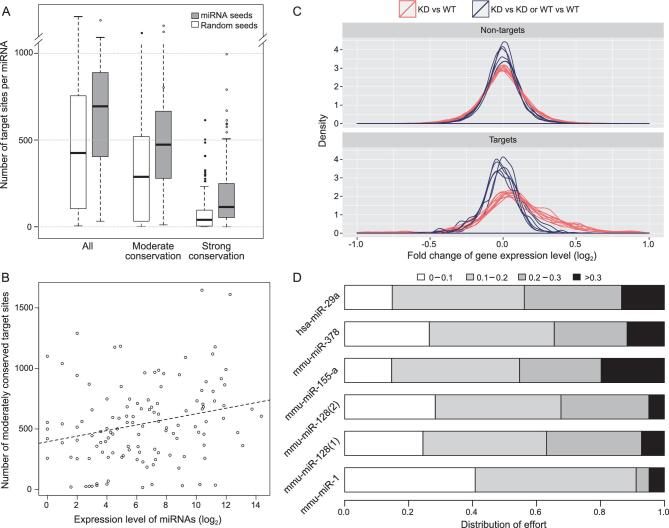
Predicted target number in relation to the observed derepression by miRNA knockout. (A) Number of miRNA target genes predicted by TargetScan (grey bars) vs. control (white bars) based on the shuffled seeds of the same miRNAs. The comparison is done at three levels of evolutionary conservation (see also Figure S1-A, B). (B) Correlation between the expression level of 109 miRNA seeds and the predicted number of moderately conserved targets. The correlation is positive but the slope is very small (see text and also Figure S1C, D). (C) Distribution of fold change in the expression of target genes in miRNA (hsp-29a) knockout lines between experiments and controls (red lines) and between controls (blue lines). The median increase upon miRNA knockout is <10%. (See also Figure S2). (D) DOE on target repression by each of 6 miRNAs. These efforts are categorized into four levels depending on the effect of repression, ranging from <10% to >30%. DOE sums up the repressions across all target genes, weighted by their expression level. Strong repression of >30% generally takes up ∼10% of a miRNA’s repression capacity.

We examined 178 conserved miRNAs in human cells for their target sites following the common protocol (Fig. 1A; see *Methods*). Random seeds with the same CG content served as the control. If all potential targets are counted, the median number of target genes would be 694, >60% higher than the control. The numbers for the moderately and highly conserved targets were 473 and 114 (64 and 185% higher than the control), respectively. While highly conserved target sites are generally considered more reliable, Xu *et al*. [30] have shown that weakly conserved targets are also evolutionarily significant. Hence, the number of targets per human miRNA is likely to be between 100 and 500 [31–33] (Fig. S1-A, B).

The large number of target sites is even more puzzling for lowly expressed miRNAs. Given their limited repression capacity, these miRNAs might be expected to have far fewer targets. Figure 1B shows the prediction to be qualitatively true. However, the slope of the regression is extremely mild with a decrease of one-third of the target number when the expression decreases by >1000-fold (Fig. S1-C, D). Hence, if only strong repressions are functional, then more than one-half of the miRNAs expressed in any tissue would be non-functional.

#### Strength of repression

With hundreds of targets, each miRNA is expected to exert weak effects on most targets. A typical example is given in Fig. 1C, which is based on six transcriptome data sets from the knockout line of hsa-29a miRNA (Fig. S2). The fold changes of target genes are symmetrically distributed around a peak that corresponds to ∼3% repression. Note that the peak is not at 0%, as is the case for non-targets. Even though hsa-29a is moderately to highly expressed, the degradation of its targets is no more than 5%, on average.

Weak repression can still result in noise as long as the weak targeting collectively does not take up much of the total capacities of mRNAs. Therefore, we measured the fraction of each miRNA’s capacity that was used in weak repression. The distribution of effort (DOE) sums up all repressions of a certain strength, weighted by the expression level of the target gene. Figure 1D shows that miRNAs use most of their repression capacity to exert small influences on a large number of target genes. Indeed, only ∼10% of the total repression capacity is used for the stronger repression (black bar, Fig. 1D). If we consider miRNAs that are themselves lowly expressed, DOE across all miRNAs would be even more biased toward weak repressions. We next analyzed weak repressions in the context of the GRN.

### GRN stability in relation to expression repression

May pointed out that large interacting systems are difficult to stabilize, contradicting the belief that large systems are inherently stable [1]. The theory may be particularly suited to GRNs because cell functions depend on transcriptome stability [34], and losing even a small number of genes can have severe consequences [35,36]. Furthermore, GRNs are periodically perturbed by cell divisions and a speedy return to equilibrium seems vital to the cells. Because the stability in transcript abundance

has been analyzed mainly at the level of small motifs [5,9], we expanded the analysis to the network level.

In a GRN with *N* genes, let *x_i_(t)* denote the mRNA concentration of gene *i* at time *t*. When the system is at an equilibrium, $\frac{{d{x_i}}}{{dt}} = \ 0$ for all *i’*s. Here, we approximated small perturbations near the equilibrium by a linear system (although the system could be non-linear globally):

(1) \begin{equation*}\frac{{d{x_i}}}{{dt}} = {B_i}\ - {D_i}{x_i}\ \left( {1 \le i \le N} \right)\end{equation*}

where ${B_i} = {b_i}\ + {S_i}$, with *b_i_* being the hypothetical transcription rate if influences of all other genes on gene *i* cancel out. Also:

(2) \begin{equation*}{S_i} = \mathop \sum \limits_{j = 1,\ j \ne i}^N {a_{ij}}{x_j}\ \end{equation*}

where *S_i_* is the aggregate effects of other genes on gene *i*, with *a_ij_* being the strength of transcriptional regulation by specific protein–DNA binding of gene *j* on gene *i*. *D_i_* is the decay rate of the mRNA of gene *i*, which would include possible autoregulation.

Following the approach of May [1] and Allesina *et al*. [2] for studying species interaction network (SIN) stability, we designated the interactions among genes by a matrix, *M*. The diagonal element, *M_ii_*, represents the effect of *x_i_* on itself and the off-diagonal element, *M_ij_*, is the regulation strength of gene *j* on gene *i*. *M* is the Jacobian matrix:

(3) \begin{eqnarray*} M\ = \left(\!\! \begin{array}{c@{\quad}c@{\quad}c@{\quad}c@{\quad}c} {\frac{{\partial {F_1}}}{{\partial {x_1}}}\left( X \right)}& & {\frac{{\partial {F_1}}}{{\partial {x_2}}}\left( X \right)} & & {\frac{{\partial {F_1}}}{{\partial {x_n}}}\left( X \right)}\\[6pt] &&& \cdots & \\ {\frac{{\partial {F_2}}}{{\partial {x_1}}}\left( X \right)}& & {\frac{{\partial {F_2}}}{{\partial {x_2}}}\left( X \right)} & & {\frac{{\partial {F_2}}}{{\partial {x_n}}}\left( X \right)}\\[6pt] & \vdots & & \ddots & \vdots \\ [6pt] {\frac{{\partial {F_n}}}{{\partial {x_1}}}\left( X \right)}& & {\frac{{\partial {F_n}}}{{\partial {x_2}}}\left( X \right)} & \cdots & {\frac{{\partial {F_n}}}{{\partial {x_n}}}\left( X \right)} \end{array}\!\! \right)\nonumber\\ \end{eqnarray*}

where:

(4) \begin{eqnarray*} {F_i}\ \left( {{x_1},{x_2}, \ldots {x_n}} \right) = {F_i}\ \ \left( {X\ } \right) = \frac{{d{x_i}}}{{dt}}\nonumber\\ = {b_i}\ + {S_i}\left( X \right) - {D_i}{x_i}\ \left( {1 \le i \le N} \right) \end{eqnarray*}

Given (3) and (4), the elements of the matrix are:

(5) \begin{equation*}{M_{ii}} = \ - {D_i}\ {\rm{and}}\ \ {M_{ij}} = {a_{ij}}\ \left( {i \ne j} \right)\!.\end{equation*}

We note that the dynamics of change in *x_i_* are linearized near equilibrium. Therefore, equations (3–7) are ‘local analysis’ as they approximate the true dynamics only in the small vicinity near equilibrium. The actual equilibrium is usually determined by empirical means, such as RNA sequencing. We first considered a network with only one gene (*N* = 1) where the stability condition is:

(6) \begin{equation*}\frac{{\partial {F_1}}}{{\partial {x_1}}}{\rm{\ }}\left( X \right) = {\rm{\ }} - {D_1} < 0.\end{equation*}

In other words, the slope of ${F_1}$ at equilibrium is negative. In this system of *N* = 1, the local analysis approximates the equilibrium as:

(7) \begin{equation*}{x_1} = \frac{{{b_1}}}{{{D_1}}}.\end{equation*}

By increasing *D*_1_ and *b*_1_ in proportion, this system could gain stability without changing the equilibrium and, indeed, the transcription and degradation have been shown to coevolve [37,38].

When *N* > 1, the stability of the system is measured in *N* orthogonal directions. The equivalent of *N* negative slopes pertaining to the stability is expressed as *N* negative eigenvalues, which is satisfied if and only if the leading eigenvalue of the matrix *M* is negative (‘eigenvalue’ in this paper only refer to the real part of eigenvalue, ‘leading eigenvalue’ is the eigenvalue have largest real part). The leading eigenvalue can be approximated as *R* – *D* [2,39]. *R*, a function of the interaction strength (i.e. the off-diagonal elements), is the leading eigenvalue of the matrix *M*_0_, which has the same off-diagonal elements as *M* but all diagonal elements are 0. $D\ = \frac{{\mathop \sum \nolimits_{i = 1}^N {D_i}}}{N}\ ( {\ = \frac{{\mathop \sum \nolimits_{i = 1}^N {M_{ii}}}}{N}\ } )$ is the average degradation rate. Therefore, the stability condition is:

(8) \begin{equation*}R - D < 0\end{equation*}

While *R* and *D* are usually obtained numerically, an analytical approximation can be derived from equation 1 of Tang *et al*. [3] when applied to actual transcription data of yeast and mammals (see later sections). Let the connectivity *r* be the proportion of non-zero *M_ij_*s $( {i \ne j} )$, and let *μ* and *σ*^2^ be the mean and variance of the non-zero off-diagonal elements. When $u \approx 0$ or < 0, and ${\rm{E}}( {{M_{ij}} \times {M_{ji}}} ) \approx 0,$

(9) \begin{equation*} R \approx \sigma \sqrt {rN} . \end{equation*}

Therefore, the stability condition is approximated by:

(10) \begin{equation*}\sigma \sqrt {rN} - D < 0.\end{equation*}

Equation (10) is suggestive of the roles of miRNAs, which increase *D* by catalytically degrading mRNAs [10]. The degradation can be expressed in two parts:

(11) \begin{equation*}-\!{D_i} = \ -\!\left( {{d_i} + {m_i}} \right)\end{equation*}

where ${d_i}$ is the basal decay constant and ${m_i}$ is the total effect of all miRNAs on the decay of gene *i*. Clearly, a larger *D_i_* would make the system more stable.

### Properties and predictions of the theory germane to miRNA functions

#### Properties

We now briefly emphasize some key features of the model. First, stability in this study means that the entire system would return to exactly the same equilibrium after small perturbations. Hence, the timescale is small for GRNs, in at most tens of minutes. At a larger timescale, the stability does not mean stasis as the equilibrium, x (x = b/D) may gradually change. For example, through stages of development, *b* may change while *D* keeps the GRN close to the equilibrium. Note that stability and change (either evolution or development) are not antithetical. Second, a stable GRN is necessary but not sufficient for biological stability. Both the proteome and metabolome downstream may have to be stabilized as well. Third, in the theory, the diagonal elements are affected by miRNAs but miRNAs themselves are not in the network, as explained below.

In mammalian cells, the total number of miRNA molecules has been reported to be in the same order of magnitude as the number of mRNAs [40,41]. Therefore, the abundance of miRNA per locus is > 100-fold greater than that of an average mRNA gene. Furthermore, the turnover of miRNAs occurs much more slowly than that of mRNAs. The half-life of

miRNAs in mammalian cells averages about 120 h in comparison with that of mRNAs at 6–8 h [42,43]. The estimates on miRNA half-lives vary partly because miRNA processing usually yields two products [44] and the minor product, so-called miR*, may be quickly degraded [45,46]. The relevant population of miRs in this study is the major product of the highly expressed miRNAs. The abundance and slow turnover of miRNAs make them nearly unchanged in the time frame of local perturbation. These features also make them uniquely suited to be the canalizing molecules.

#### Predictions

The theory makes several predictions, which provide a unified perspective on a suite of miRNA properties that have only been explained individually. One key property in fact has never been explained.

##### Broad distribution of the degradation effect of miRNAs: how broad?

The theory shows that the average degradation, $D\ = \frac{{\mathop \sum \nolimits_{i = 1}^N {D_i}}}{N}\ $, or the total degradation, *D_T_ = D × N*, is a main determinant of GRN stability. This property does not suggest how *D_T_* should be distributed. In metazoan cells, the total repression effect is distributed among many miRNAs and the effect of each miRNA is further distributed over hundreds of target genes. Qualitatively, the total repression of miRNAs is diffusive over the entire GRN.

**Figure 2.**
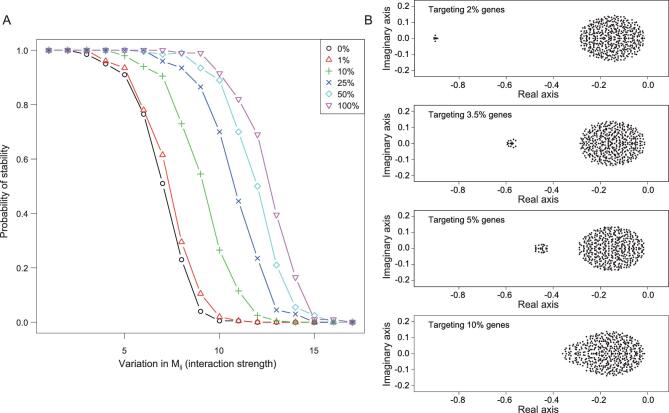
GRN stability in relation to the spread of total miRNA repression in the GRN. (A) The *y*-axis is the probability of GRN stability, determined by the proportion of cases yielding a negative leading eigenvalue in 200 simulations. The *x*-axis is the variation in interaction strength presented as the (relative) standard deviation of *M_ij_*. The repression is distributed over 1–100% of the entire GRN. While the total repression is constant, the probability of stability increases when the effect is spread more broadly over the network. The increase is most rapid from 1–25% and slows down gradually. (B) Distributions of eigenvalues as miRNA targeting becomes more diffuse. If the repression is concentrated on a few genes, only a small fraction of eigenvalues is affected, shown by the outliers on the left. Neither the bulk of the distribution nor the leading eigenvalue is noticeably changed. Only when the targeting is sufficiently broad would the entire distribution shift to the left, thus dragging along the leading eigenvalue.

Here, we provide a quantitative evaluation of various distributions of miRNA targeting while keeping their aggregate effect constant, at 10% of the total (i.e. $\mathop \sum _{i=1}^N {m_i} = {\rm{\ }}10{\rm{\% }}\mathop \sum _{i=1}^N {D_i}$). Figure 2A shows that, given the same network complexity, the GRN is more likely to be stable when miRNA targeting becomes more diffuse (i.e. targeting more genes with less intensity; see *Methods*). When only 1% of the mRNAs are targeted for repression by all miRNAs, the probability of GRN stability is only slightly higher than a GRN without any repression. On the other hand, when the repression already covers 25% of all transcripts, further spread would have only incremental benefits on the stability.

An intuitive explanation is illustrated in Fig. 2B. When miRNAs target a small percentage of genes, only a few eigenvalues are affected and shifted very far to the negative side. The leading eigenvalue is hardly affected, hence resulting in only marginal improvement in GRN stability. The more diffuse the targeting, the more eigenvalues are shifted to the left, eventually dragging the leading value down. Estimates of miRNA targeting fall in the range of 25–60% of all mRNAs in human cells [31,32], in reasonable accord with the theoretical prediction of >25%. The next section will explore whether targeting is randomly distributed among all mRNAs.

##### Avoidance of very highly expressed mRNAs.

**Figure 3.**
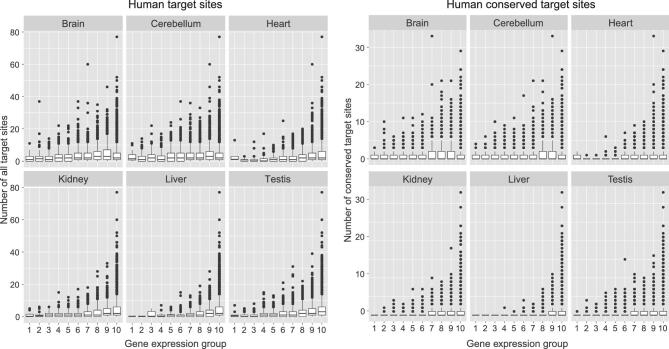
Number of miRNA target sites on genes with different levels of expression, ranging from high to low from left to right in 10 different groups, each containing 10% of all genes. The left sets of panels are analyses of all targets of 109 miRNA seeds, and the right sets of panels are those of conserved targets. Analyses of two different levels of evolutionary conservation are shown but the pattern is observable in all (see Supplementary Data). For each level, six tissues are analyzed. Note that very highly expressed genes appear to avoid having a very large number of target sites. (See also Figure S3).

Another aspect is the expression levels of miRNA targets. In this theory, miRNAs are expected to avoid targeting very highly expressed genes. Given a fixed repression capacity, targeting the most highly expressed genes is a wasteful strategy for two reasons. First, highly expressed genes may act as ‘sponges’ [47], soaking up many miRNAs and leaving few available for other less highly expressed targets. Second, high abundance transcripts should be less affected by stochastic fluctuations; for example, after cell divisions. This prediction is supported by empirical data. Figure 3 is a typical example in which relatively few highly expressed genes have a high number of target sites (Fig. S3).

##### Preference for transcription factor targeting.

Given the collective targeting by miRNAs, almost all classes of genes are their targets. In the entire GRN, transcription factors (TFs) are the well-known class of targets preferred by miRNAs and there are many explanations [48–51]. Here, the GRN theory offers a new perspective based on the hierarchical structure within the network. Since TFs constitute a higher level and highly connected sub-network, the theory would predict TFs to be preferentially targeted by miRNAs. A detailed presentation is given in the Supplemental text where various explanations are compared.

### Comparisons of GRNs with and without miRNAs

An alternative approach to the function of miRNAs is to compare GRNs with and without miRNAs. We used human GRNs for the former and yeast GRNs for the latter. While the theory of network stability (Eqs. 8–10) requires sufficient repressions of mRNAs, there are other solutions besides the actions of miRNAs. In fact, repressing mRNAs non-specifically via RNases (as yeast cells do [52]) could be a more powerful means to confer GRN stability. The comparison may reveal different strategies of GRN stabilization and shed further light on the functionality of miRNAs. In comparing human and yeast GRNs, we estimated the diagonal and off-diagonal elements of the interaction matrix *M* separately, and then put them together to compare their stability.

#### Degradation (the diagonal elements)

**Figure 4.**
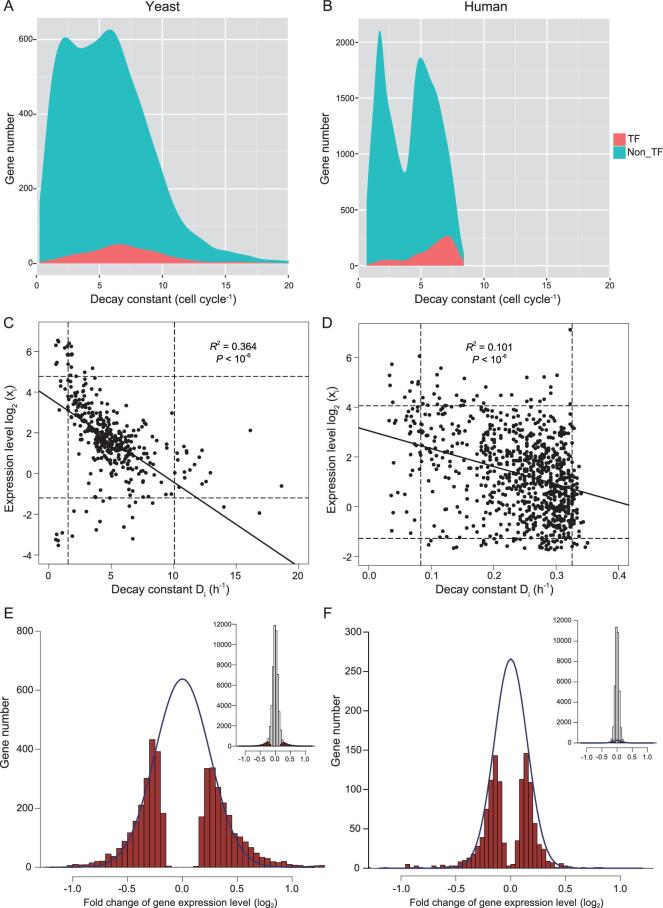
Measurements of degradation and interaction in GRNs. (A, B) Density plot of *D_i_* (mRNA decay constant). TFs (shaded in red) have higher degradation rates than the rest of the transcriptome (blue). The rates shown are calibrated by the respective cell cycle time, by which the two systems are comparable in degradation. In actual time, yeast mRNAs are degraded 17 times faster than human mRNAs. (C, D) The relationship between the expression level of mRNAs and the decay constant. The *y*-axis spans three orders of magnitude while the *x*-axis spans only one order. Hence, the expression level of genes is only marginally affected by the degradation constant. The dotted lines mark 5% and 95% of the distribution. In this restricted range, Y also varies more than X by 10 fold. (E, F) Distribution of the interaction strength between genes in yeast and human GRN. The strength is the change in the abundance of mRNA of gene *i* upon the knockout (yeast) or knockdown (human) of gene *j*. Significant changes (*P* < 0.001) are marked in red and approximated by a normal distribution marked by the blue line. The inset displays this portion of significantly changed genes relative to all genes.

The degradation rates of transcripts in many GRNs have been measured, usually by turning off thetranscription and monitoring the decay of mRNAs [53,54]. It is known that the mean half-life for yeast mRNAs is ∼15 min [55], whereas it is 4–8 h for human mRNAs [43]. Figure 4A and B show that the median decay constant (measured in molecules/hour) for yeast mRNAs is 17.4 times larger than that for human mRNAs. Interestingly, when calibrated against their respective cell doubling times of 1.5 and 24 h, the degradation rates are roughly equal (6.87 vs. 6.33) for yeast and human transcription factors (TFs, red shades of Fig. 4A and B).

One might expect the variation in degradation to be driven by natural selection to fine-tune the expression level, *x_i_*. The regression of *x_i_* over *D_i_* is indeed significantly negative for yeast and human genes, but the correlation coefficient is small (*R^2^* = 0.364 and 0.101 for yeast and human, respectively; Fig. 4C and D). Importantly, this is not a simple inverse relationship, as *x_i_* spans three orders of magnitude and *D_i_* varies by only one order (Fig. 4C and D). Even when we exclude the tails of the distributions (5% on either end), *x_i_* still varies 10-fold more than *D_i_*.

Therefore, the variation in expression is largely due to the variation in synthesis rather than degradation (see Supplement on *D_i_* variation). If the many cellular components, including miRNAs and RNA-binding proteins, that function in mRNA degradation do not set the level of gene expression, the question is then ‘what roles may mRNA degradation play in the GRN?’.

#### Strength of gene interaction (the off-diagonal elements)

Figure 4A and B show that *D* is much larger in the yeast GRN than in human. Since the smaller *R–D* is, the greater the stability becomes (Eq. 8), yeast GRN could be much more stable than human GRN. Alternatively, yeast GRN might have a correpondingly larger *R* (Eq. 9) and the two GRNs would be comparably stable. We hence analyzed the measurements of *M_ij_* based on experiments that delete or suppress the expression of one TF at a time [56,57]. These two databases are used for both biological and technical reasons, and they are also the largest publicly accessible databases. Nevertheless, the conclusion does not depend on the actual databases used. The TF sub-network is most responsible for the stability of the entire GRN, given its higher position within the hierarchy (see below). The effects of TF deletion/suppression are assayed by transcript analysis (see *Methods* and the Supplemental text for further explanations).

We now describe the construction of the yeast GRN. In order to determine the proper size of the network, we ranked genes by their expression in descending order. The set of the most highly expressed TFs with *N* = 356 collectively accounted for 99% of total mRNAs. *N* = 356 is the size of the yeast GRN. The procedures for estimating the regulation strength have been widely reported. Several were used here [56,57] (see *Methods*).

The distribution of the estimated regulation strength is given in Fig. 4E and its inset. Among all interactions, 4234 are significant with *P* < 0.001, yielding a connectivity of *r* = 0.076. Figure 4E shows the significant regulations with red bars, which are approximated by a normal distribution. The non-significant regulations are set to 0. The normal distribution containing all significant interactions is shown relative to the entire set in the inset. In summary, positive:negative regulation is evenly split with a 0.504:0.496 ratio. The mean (*μ*) and standard deviation (*σ*) are 0.0144 and 0.432 (see legends). The mean and standard deviation of the absolute value of the interaction strength (|*M_ij_*|) are, respectively, 0.379 and 0.207. We note that, in order to construct the GRN, we estimate the mean, variance and distribution of *M_ij_*. The identities of specific nodes that are connected are not crucial in determining the identify. This aspect of GRN in relation to miRNA function will be discussed.

**Figure 5.**
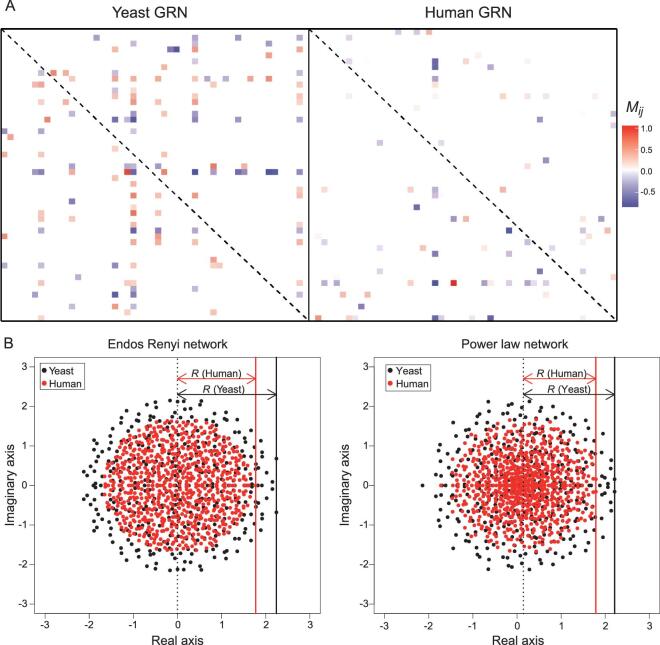
The interaction matrix (*M*) and its eigenvalues. (A) A random 50 × 50 block of the interaction matrix, *M*, is shown for the yeast and human GRNs. Off-diagonal elements that are significantly positive or negative (see Fig. 4E and F) are indicated by a color of the heatmap. Diagonal elements are not shown (marked by the dashed line). Note that the yeast GRN is slightly denser than the human GRN. (B) Distribution of the eigenvalues of GRNs, which are complex numbers with real and imaginary parts. The GRN is constructed with the parameters obtained from the measurements of Fig. 4, with the off-diagonal elements following a normal distribution (Fig. 4E and F) and the diagonals set to zero. Two different network structures (random network and power-law) are modeled but the outputs are similar. The leading eigenvalue, marked by a solid vertical line, has a value of *R* (see equations (8) and (10)). The GRN stability requires that *R–D* < 0.

In the human GRN, the corresponding *M_ij_* distribution is shown in Fig. 4F where *N* = 746 and *r* = 0.031. The positive:negative split is 0.53:0.47, *μ* = –0.0322 and *σ* = 0.244. The mean and standard deviation of |*M_ij_*| are 0.195 and 0.151 (see *Methods*). A presentation of a small portion (50 × 50) of each of the two GRNs is shown in Fig. 5A. The comparison visually portrays the difference in connectivity between human and yeast GRNs (*r* = 0.031 vs. *r* = 0.076) with the latter being denser. Therefore, while the human GRN is larger than that of yeast (*N* = 746 vs. *N* = 356), the number of connections per node, $\frac{{{N^2}r}}{N}$, is very similar with *Nr* = 23.1 vs. 27.1. The effective sizes [58] are hence similar between the two GRNs. The interaction strength in the human network appears weaker, but only mildly so.

In both networks, significant regulations are not randomly distributed among nodes as a small fraction of nodes are disproportionately more connected than the rest [59,60]. Figure S4 presents the distribution of in-degree connections, or the number of significant regulations going toward a node as required in (2). The observed distribution is closer to the power-law than to random distribution, corroborating previous analyses [59,60].

#### GRN stability in yeast vs. human: joint effects of diagonal and off-diagonal elements

With the off-diagonal *M_ij_* values, the eigenvalues of the matrix *M*_0_ can be determined as shown in Fig. 5B for yeast (black dots) and human (red dots) GRNs. Note that the diagonal elements of *M*_0_ are set to 0, in comparison with *M* of (3). Marked by a vertical line in Fig. 5B, *R* roughly corresponds to the ‘radius’ of the eigenvalue distribution. The two panels of Fig. 5B also show that the eigenvalue distributions are not noticeably changed by the network structure (random vs. power-law interactions).

The *R* values in yeast and human GRNs differ very slightly (2.2 vs. 1.8 in Fig. 5B) over a wide range of cut-offs used in the estimation. Given that *R* is similar and *D* is 15 times larger in yeast, *R–D* is much more negative in yeast than in human. In other words, the yeast GRN is much more stable than the human GRN; thus, when perturbed, the yeast GRN should be able to return to equilibrium much more rapidly. Interestingly, yeast cells can divide 15 times faster than human cells (1.5 h vs. 24 h) and, hence, would be perturbed more frequently.

## DISCUSSION

### Comparative strategies of canalization

We applied the May–Wigner theory to two very different GRNs, one with and the other without miRNAs. The yeast GRN may be able to use a simpler strategy for GRN stabilization because unicellular organisms do not have different tissues with different cellular properties. Furthermore, because a typical haploid yeast cell is only 1% as large as an average-sized human cell [61,62], the transcription rate per unit volume can be much higher in yeast than in human cells. For these reasons, the yeast GRN may be able to have non-specific degradation of transcripts that is as rapid as transcription can keep up. This simple strategy would be neither feasible nor necessary for human GRNs. In metazoans, GRNs may need to adjust the strategy of stabilization in different cell types. Their larger cell volume also demands far more transcripts; thus, a high rate of mRNA degradation may stress the supply to a much larger degree. A suitable strategy for mammals would be to degrade mRNAs more selectively and modestly, and miRNAs might have evolved for these reasons.

### Canalization by miRNAs in metazoans

The pervasive weak action of miRNAs has been a contentious issue, giving rise to the view that most targets are biologically irrelevant [17,18,44,63–67]. Since the sum of weak repressions accounts for >90% of the total activities of miRNAs, it is difficult to reconcile this view with a simple calculation. Instead, the May–Wigner theory suggests that weak repressions can cumulatively contribute to GRN stability [17]. Furthermore, the more diffuse the repression effect, the more stable the network.

In animals, miRNAs may be the true system-level regulators. It is their wiring pattern, rather than specific links between genes, that is germane to their function. Importantly, by stabilizing GRNs, miRNAs would stabilize the downstream phenotype, albeit indirectly. These molecules are hence the likely agents of developmental canalization proposed by Waddington >60 years ago [7,8,68–70]. A recent study [18] found that miRNAs often control the same phenotypes incoherently through multiple target genes. Incoherent control loops are usually associated with stasis rather than change [11–14]. Finally, the contrast between the diffuse actions of miRNAs in animals and the more concentrated repressions in plants [71,72] raises interesting questions about the divergence between plants and animals in relation to the ancestral functions of miRNAs.

*Caveats* - The theory presented here is a sharp break from the conventional views on miRNA functionality. Naturally, caveats need to be heeded. In the first section of the Supplement, most common objections, as well as our rebuttals, are given.

## METHODS

### Number of targets

Target sites of 178 conserved miRNA’s from eight closely related species were predicted in the human genome by seed matching using TargetScan (targetscan.org). Seed matches better than 7mer were considered target sites. The associated random seeds with the same CG content serve as the control. Target sites are stratified by conservation into three levels: all target sites, moderately conserved target sites and strongly conserved target sites (Table S2).

Similar analyses were applied to 94 conserved miRNAs in fly and 167 conserved miRNAs in mouse. Conserved miRNA family lists and untranslated regions (UTRs) were downloaded from TargetScan.org. The numbers of conserved miRNA families and 3′-UTRs are shown in Table S1. Data of miRNA expression come from Landgraf *et al*. and Lyu *et al*. [73,74].

### Strength of repression

To measure the regulation strength of single miRNAs, we examined the change of transcriptome after single miRNA knockout (or knockdown). The transcriptome data of knockout or knockdown lines were downloaded from the Gene Expression Omnibus database. We collected ∼50 data sets to examine their repeatability and selected 5 high-quality (correlation between replication > 0.99) data sets for further analysis (Table S3). The microarray data were processed by the Bioconductor package. To measure the strength of miRNA repression, we calculated the pairwise fold change of each sample, and separated the fold changes into two categories: (i) between an miRNA-deficient transcriptome and a wild-type one, and (ii) between two miRNA-deficient transcriptomes or two wild-type ones. These two categories of fold changes were directly compared by density plot.

DOE sums up all repressions of a certain strength, weighted by the expression level of the target gene. These efforts were categorized into four levels depending on the effect of repression, ranging from <10% to >30%. DOE sums up the repressions across all target genes, weighted by their expression level. Strong repression of >30% generally takes up ∼10% of a miRNA’s repression capacity.

### Simulations of the degradation effects of miRNAs

Random matrices were generated with the parameters *N* = 500, *r* = 0.1, ${M_{ij}}( {i \ne j} )$∼*N*(0, *a*) and *d* = 0.1. The value *a* of *N*(0, *a*) increases from 0.1275 to 0.1675. The probabilities of stability were calculated from 200 simulations. We plotted four cases to illustrate the influence of target numbers on the eigenvalues (Fig. 2A and B).

### Decay rate

The mRNA half-life data were downloaded from public data [43,55], which contained the half-life measurements for 5656 yeast mRNAs and 10 290 human mRNAs. We transformed the half-life measurements into the decay rate using the exponential model:

$$\begin{equation*}
d \, = \frac{{ln2}}{t}
\end{equation*}$$

where *d* is decay constant in units of hours^−1^ and *t* is the half-life in units of hours.

For further comparisons, the decay constants of yeast and human mRNAs were shown as the value normalized by the cell cycle time (1.5 h for yeast and 24 h for human), in units of cell cycle^−1^.

### Strengths of gene interactions

To measure the strength of gene–gene transcriptional regulation, we examined the change of transcriptome after single TF knockout or knockdown. The normalized transcriptome data were downloaded from public data [56,57]. The fold changes between the control and TF-deficient transcriptome were calculated with corresponding *P* values. Significant transcription regulations were defined by *P* < 0.001. We represented gene–gene interaction strength (*M_ij_*) with fold changes for significantly differential expressions.

### Construction of matrix *M*

The size of the matrix, *N*, is determined by the number of TFs that in aggregate account for 99% of the total transcripts. We generated two kinds of network: an Endos Renyi network and a power-law network, using R package(igraph) in order to determine the non-zero elements in the matrix. The values of non-zero elements in the network were sampled from the gene–gene regulations estimated above. The eigenvalues were calculated and plotted in the complex plane (Fig. 5B).

### Considerations of selective pressure

The arguments presented in this study are mechanistic in nature. For biological systems, these arguments have to incorporate selective forces as is done explicitly in other studies [75–77]. These considerations also underlie the overall approach of this current study.

## Supplementary Material

nwz076_Supplemental_File
