# Supplementary material for: Gene regulatory network stabilized by pervasive weak repressions: microRNA functions revealed by the May–Wigner theory
Source: Natl Sci Rev. 2019 Jul 25;6(6):1176–88. doi: 10.1093/nsr/nwz076 (PMC8291590; doi:10.1093/nsr/nwz076)
Supplement: nwz076_Supplemental_File [file nwz076_supplemental_file.pdf]

Supplemental Information

Preference for TF targeting

In this study, we focus on the sub-network of TFs because GRNs have a hierarchical structure in which nodes of the higher rank regulate those of the lower rank, but not vice versa. The hierarchical structure would have strong influences on how miRNAs "choose" their targets because a hierarchical network can be decomposed into sub-networks in stability analysis (Stewart, 2001). For GRNs, TFs form a sub-network at a higher hierarchy above non-TFs and are more highly connected (Spitz and Furlong, 2012; Stampfel et al., 2015), which make sub-network of TFs is less stable. Thus, the stability of the GRN would be strongly dependent on the stability of the TF sub-network (Fig. S5-A). It is therefore expected that miRNAs would preferentially target TFs. Indeed, the most conspicuous category of genes significantly enriched for miRNA targets are TFs (Chen et al., 2011; Croft et al., 2012; Cui et al., 2006; Dannemann et al., 2012). In our re-compilation of miRNA targets in fly, mouse and human, TFs are enriched over the rest of the transcriptome by 16.8%, 15.1%, 13.3%, respectively. If we consider only conserved target genes, the enrichment becomes 89.2%, 49.6% and 41.2% (Fig. S5-B). While TFs have been known to be miRNAs' preferred targets (Chen et al., 2011; Croft et al., 2012; Cui et al., 2006; Dannemann et al., 2012), the reasons vary and some (Chen et al., 2011; Croft et al., 2012; Cui et al., 2006; Dannemann et al., 2012) are implicitly based on the network structure.

References

Chen, C.Y., Chen, S.T., Fuh, C.S., Juan, H.F., and Huang, H.C. (2011). Coregulation of transcription factors and microRNAs in human transcriptional regulatory network. Bmc Bioinformatics *12*.

Croft, L., Szklarczyk, D., Jensen, L.J., and Gorodkin, J. (2012). Multiple independent analyses reveal only transcription factors as an enriched functional class associated with microRNAs. BMC Syst Biol *6*, 90.

Cui, Q.H., Yu, Z.B., Purisima, E.O., and Wang, E. (2006). Principles of microRNA regulation of a human cellular signaling network. Molecular systems biology *2*.

Dannemann, M., Prufer, K., Lizano, E., Nickel, B., Burbano, H.A., and Kelso, J. (2012). Transcription factors are targeted by differentially expressed miRNAs in primates. Genome Biol Evol *4*, 552-564.

Spitz, F., and Furlong, E.E.M. (2012). Transcription factors: from enhancer binding to developmental control. Nature Reviews Genetics *13*, 613-626.

Stampfel, G., Kazmar, T., Frank, O., Wienerroither, S., Reiter, F., and Stark, A. (2015). Transcriptional regulators form diverse groups with context-dependent regulatory functions. Nature *528*, 147-+.

Stewart, G.W. (2001). Matrix Algorithms Volume II: Eigensystems.

Table S1. (cited in Figure 1). Numbers of Conserved miRNA families and 3'UTRs

Species	Conserved miRNA family	3'UTR	Gene
**D.melanogaster**	94	14094	11265
**Mus musculus**	167	23825	18461
**Homo sapiens**	178	30888	19919

Table S2. (cited in Figure 1 and Figure S1). Conservation stratification

Species	Taxa that define moderate conservation	Taxa that define strong conservation
**D. melanogaster**	Melanogaster subgroup	Sophophora
**Mus musculus**	Rodents	3 other mammalian order
**Homo sapiens**	Primates	3 other mammalian order

Table S3. Related to Figure 1C, Figure S2. Data for miRNA regulatory strength analysis

Species	miRNA family	NCBI ID	Platform	Tissue	Experiment
**Mouse**	miR-1-2	GSE7333	GPL1261	Heart	Knock out
**Mouse**	miR-128	GSE48813	GPL1261	Neurons	Knock out
**Mouse**	miR-155	GSE44649	GPL6246	T cell	Knock out
**Mouse**	miR-378-3p	GSE34873	GPL6246	NIH-3T3 fibroblast	Knock out
**Human**	miR-29a	GSE45564	GPL6244	Dermal fibrablast	Inhibitor

Supplementary Figure

Figure S1

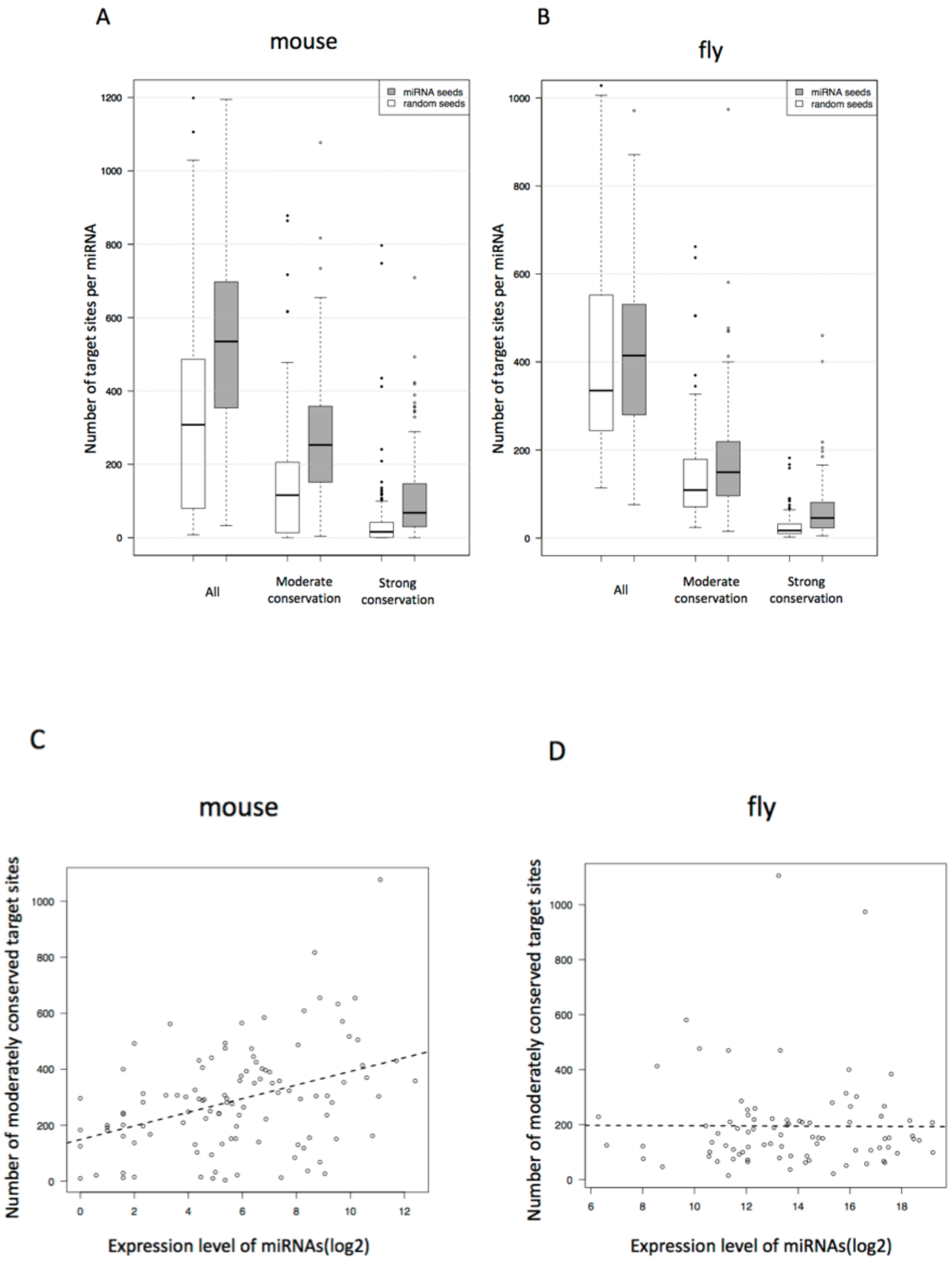

Figure S1. Related to Figure 1AB. Predicted target number

(A, B) Number of miRNA target genes predicted by Targetscan (grey bars) vs. control (white bar) based on the shuffled seeds of the same miRNAs. The comparison is done at three levels of evolutionary conservation.

(C, D) Correlation between the expression level of miRNAs seeds and the predicted number of moderately conserved targets. The correlation is positive but the slope is very small in mouse, and there is no correlation in fly.

Figure S2

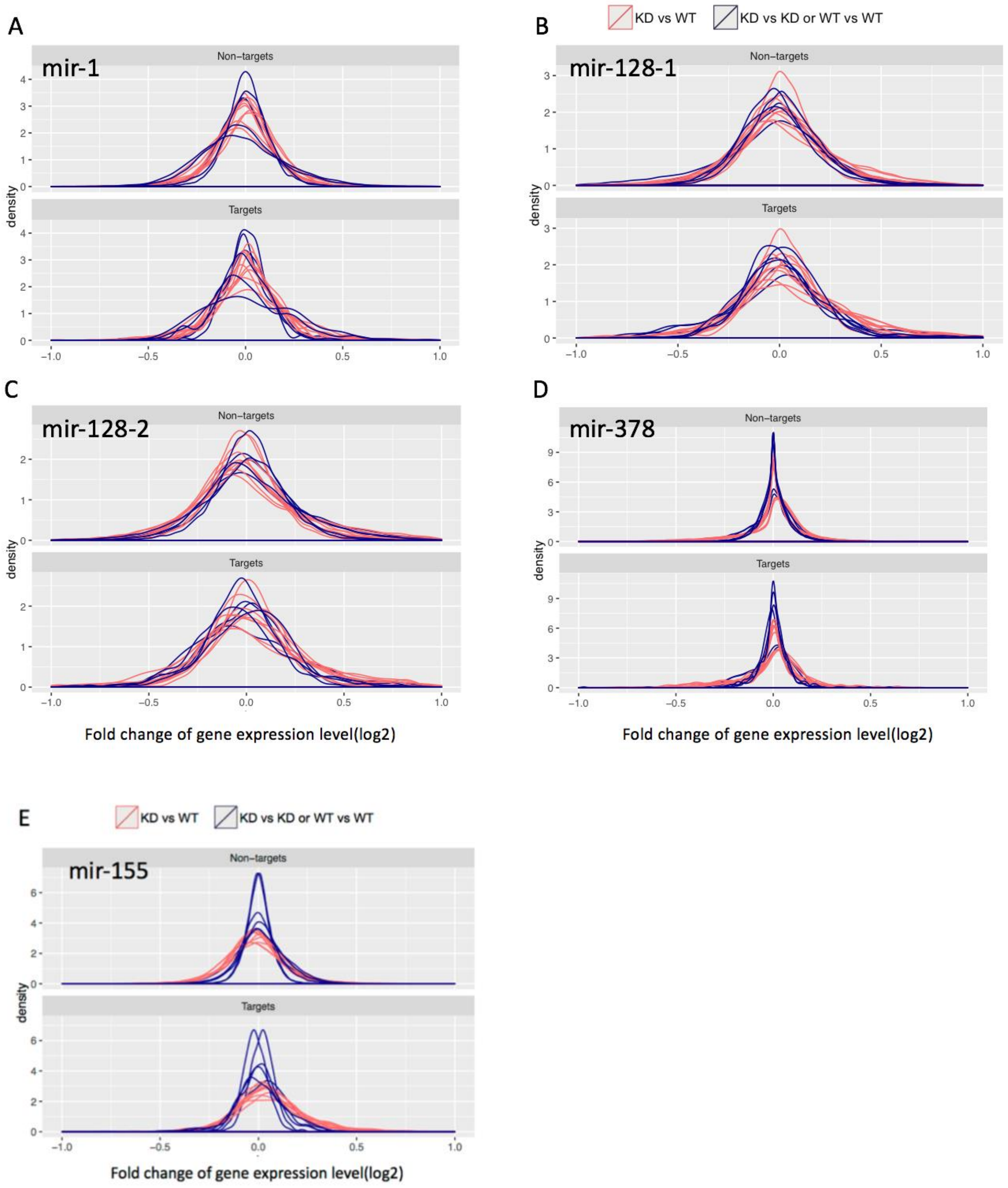

Figure S2. Related to Figure 1 C. observed de-repression by miRNA knockout
Distribution of fold change in the expression of target genes in 5 miRNA knockout lines between experiments and controls (red lines) and between controls (blue). The median increase upon miRNA knockout is < 10%.

Figure S3

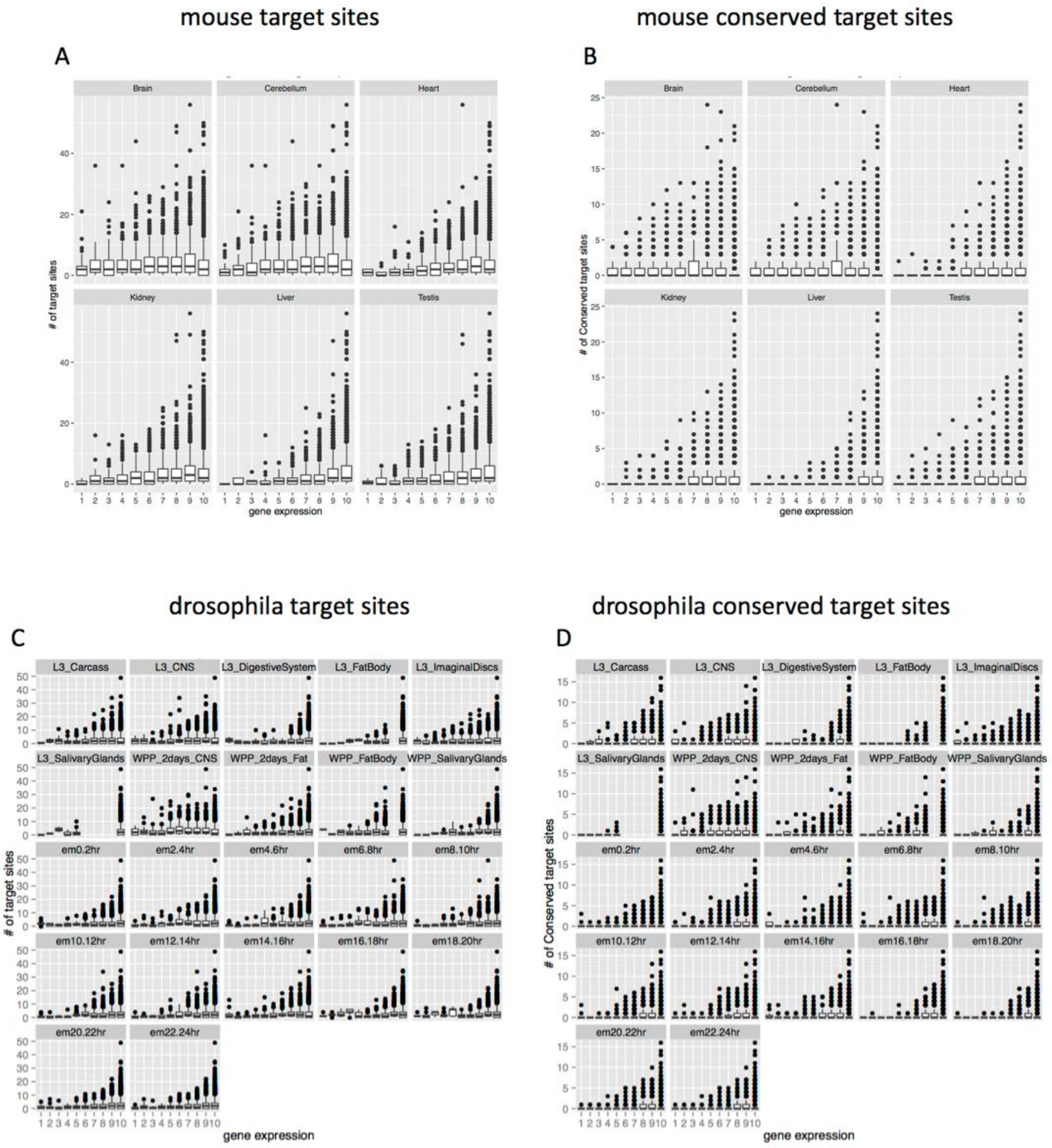

Figure S4. Related to Figure 5. Number of miRNA target sites on genes with different levels of expression, ranging from high to low from left to right in 10 different groups, each containing 10% of all genes. The left set of panel are analysis of all targets, the right set of panel are that of conserved targets. For each level, 6 tissues of mouse, 22 tissues of fly were analyzed. Note that very highly expressed genes appear to avoid having a very large number of target sites.

Figure S4

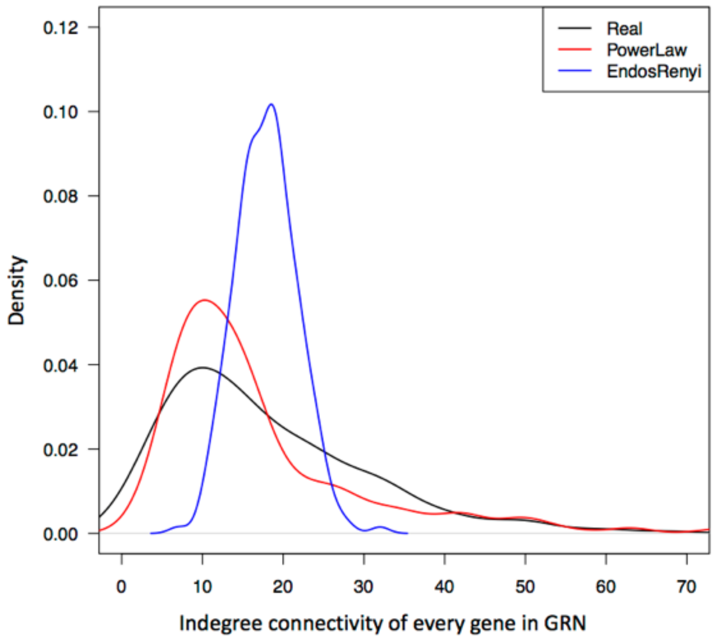

Figure S3. The distribution of the in-degree connectivity (i.e., the number of genes that significantly influence the expression of each gene) in the yeast data, marked in black. Simulations using the power-law or random (Endos-Renyi) distribution are also shown. The observed distribution of yeast-GRN connectivity is closer to the power-law distribution.

Figure S5

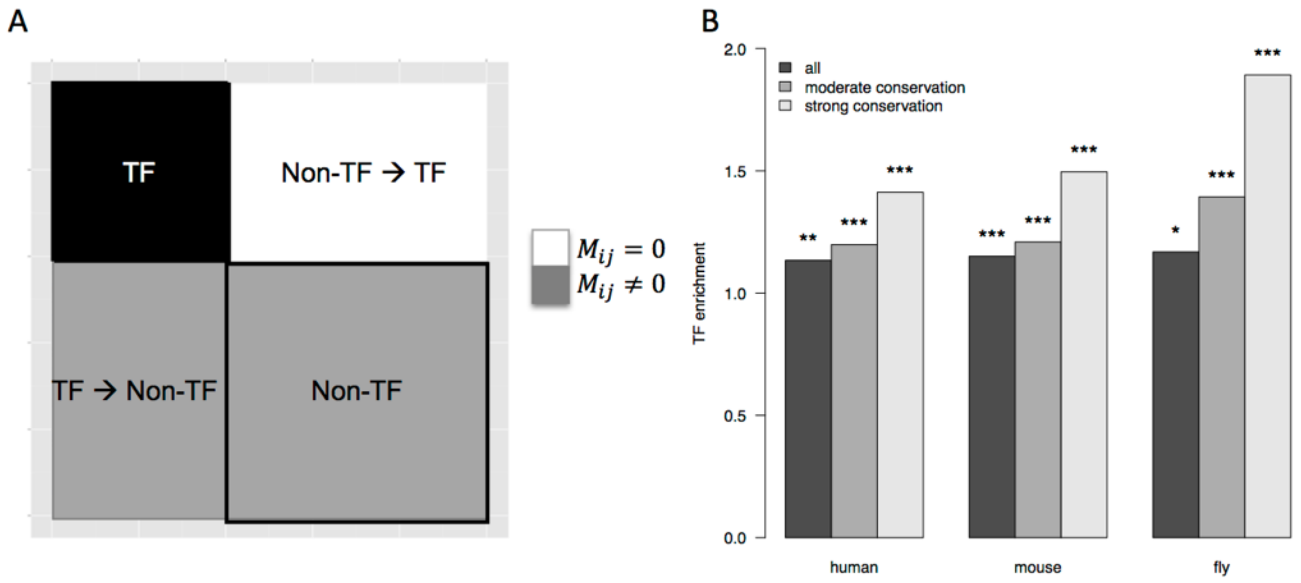

Figure S5 TF enrichment

(A)GRN have a hierarchical structure in which nodes of the higher rank(TFs) regulate those of the lower rank(non-TFs), but not vice versa. Eigenvalues of such networks can be decomposed into sub-networks at different hierarchies.

(B)Number of TF with miRNA target sites were compared to number of miRNA targets, and total TF number and total gene number served as control. Chi-square test were performed to determine the enrichment, and noted as stars. (*: $P < 0.05$, **: $P < 0.01$, ***: $P < 0.001$). Similar analysis was applied to human, mouse and fly.
